# Physiological, Structural, and Functional Insights Into the Cryoprotection of Membranes by the Dehydrins

**DOI:** 10.3389/fpls.2022.886525

**Published:** 2022-04-28

**Authors:** Marijke R. Murray, Steffen P. Graether

**Affiliations:** ^1^Department of Molecular and Cellular Biology, University of Guelph, Guelph, ON, Canada; ^2^Graduate Program in Bioinformatics, University of Guelph, Guelph, ON, Canada

**Keywords:** dehydrins, membranes, abiotic stress, physiological response, intrinsically disordered proteins, structure

## Abstract

Plants can be exposed to cold temperatures and have therefore evolved several mechanisms to prevent damage caused by freezing. One of the most important targets are membranes, which are particularly susceptible to cold damage. To protect against such abiotic stresses, plants express a family of proteins known as late embryogenesis abundant (LEA) proteins. Many LEA proteins are intrinsically disordered, that is, they do not contain stable secondary or tertiary structures alone in solution. These proteins have been shown in a number of studies to protect plants from damage caused by cold, drought, salinity, and osmotic stress. In this family, the most studied proteins are the type II LEA proteins, better known as dehydrins (dehydration-induced proteins). Many physiological studies have shown that dehydrins are often located near the membrane during abiotic stress and that the expression of dehydrins helps to prevent the formation of oxidation-modified lipids and reduce the amount of electrolyte leakage, two hallmarks of damaged membranes. One of the earliest biophysical clues that dehydrins are involved in membrane cryoprotection came from *in vitro* studies that demonstrated a binding interaction between the protein and membranes. Subsequent work has shown that one conserved motif, known as K-segments, is involved in binding, while recent studies have used NMR to explore the residue specific structure of dehydrins when bound to membranes. The biophysical techniques also provide insight into the mechanism by which dehydrins protect the membrane from cold stress, which appears to mainly involve the lowering of the transition temperature.

## Introduction

The plasma membrane is a primary site of damage resulting from stressors such as cold, drought, and osmotic stress. Each of these stressors cause a host of structural and compositional changes to the membrane lipids (Gupta et al., 2016). In this review, we mainly focus on the damage effects caused by low temperatures, and the evidence that dehydrins are involved in preventing that damage. During periods of cold temperatures, the degree of unsaturation lipids in the cell membranes increases. Conversely, during warm temperatures, the membrane lipids become more saturated (Murata and Los, 1997). This changes the membrane fluidity, which induces a conformational change in many transmembrane and peripheral proteins. Membrane receptors then respond to the stress-induced change by initiating signaling pathways that influence gene expression related to stress tolerance (Kosová et al., 2015). This initial stress response is called the alarm phase and involves several proteomic and chemical changes to the cell environment, including phosphorylation of phosphoinositides (PIs) and enzymes in their metabolic pathways (Kosová et al., 2015).

Despite the desaturation of membrane lipids, there is still the danger of cold stress inhibiting normal membrane function (Steponkus, 1984). For example, dehydration caused by freezing temperatures can result in a phenomenon known as interbilayer fusion, which occurs between the plasma membrane and other intracellular membranes, such as mitochondrial, chloroplast, or Golgi membranes (Welti et al., 2002). At temperatures from −4°C to −10°C, membranes can undergo lamellar to hexagonal (II) phase transitions (Welti et al., 2002). This phase transition is a shift from a typical lipid bilayer to an inverted micelle structure with the fatty acid tails facing outward (Jouhet, 2013). Although the lamellar to hexagonal (II) phase is reversible, both of these occurrences can have serious effects on plant health and survival.

## Dehydrin—A Plant Stress Response Protein

One protective method that plants have evolved to combat this damage involves the expression of proteins known as dehydration-induced proteins (dehydrins). Dehydrins are a group of proteins expressed in plants to protect them from damage caused by dehydration stress (mainly drought, cold, salinity, and osmotic stresses; Graether and Boddington, 2014; Aziz et al., 2021; Riyazuddin et al., 2021; Smith and Graether, 2022). Dehydrins were originally identified as being group II proteins of the late embryogenesis abundant (LEA) proteins, a large family of proteins that are expressed in response to abiotic stress. LEA proteins were first recognized as being overexpressed in seeds (Galau et al., 1986; Roberts et al., 1993), and they have since been found to be expressed under abiotic stress conditions in adult plants as well. The presence of dehydrins has been established in the genomes of all embryophytes (Malik et al., 2017; Nagaraju et al., 2018), suggesting that they have been around at least since the emergence of early land plants, and may possibly predate them (Li et al., 1998).

Dehydrins are defined by the presence of one or more lysine-rich, semi-conserved motifs known as K-segments (Close, 1997). Previous work (Malik et al., 2017) has shown that the K-segment can be defined as [XKXGXX(D/E)KIK(D/E)KXPG], where X can be any amino acid. The conservation of the lysine residue positions suggests that this amino acid plays a key role in dehydrin function, which was shown to be true for membrane protection (described below).

In addition to the K-segment, dehydrins also may contain three other motifs known as the Y-, F-, and S-segments. The motifs are present a variable number of times in dehydrins, resulting in several different architectures, specifically YSK_n_, FK_n_, FSK_n_, SK_n_, YK_n_, K_n_, and K_n_S (Smith and Graether, 2022). In between, the conserved motifs are what are known as ϕ-segments; poorly conserved regions that mainly consist of small amino acids and are highly variable in length. Another conserved motif in dehydrin is the S-segment. As the name implies, this motif contains serine residues and can be described as [LHR(S/T)GS_4-6_(S/D/E)(D/E)_3_] (Malik et al., 2017). Two possible roles have been identified for this segment, both of which involve phosphorylation of the serines (Goday et al., 1994; Alsheikh et al., 2003). One study showed that the phosphorylation helps promote localization of the dehydrin to the nucleus (Goday et al., 1994), where it may be able to protect DNA and nuclear proteins. Another suggests that they are involved in the binding of calcium ions, which the authors speculate may allow the dehydrin to buffer the intracellular calcium concentration (Alsheikh et al., 2003).

The Y-segment, which can be described as [D(D/E)(Y/H/F)GNPX], where the X is often a hydrophobic amino acid, was originally named after the conserved tyrosine residue in the middle of the motif. Further analysis shows that this position may instead contain histidine or phenylalanine. Note that tryptophan, while aromatic is not found at this position, likely because compared to other aromatic amino acids, it promotes folding (Barua et al., 2008; Ghanmi et al., 2022). The F-segment ([EXXDRGXFDFX(G/K)]) is a relatively recently identified motif (Strimbeck, 2017; Riley et al., 2019). Similar to the Y-segment, it is named after the presence of two conserved aromatic amino acids, namely, phenylalanine. The biological roles of these two motifs have not yet been fully identified. Previously, it was speculated that the Y-segment may bind ATP, but this was shown to not be correct (Boddington and Graether, 2019).

Dehydrin sequences are rich in polar and charged amino acids, and therefore, it is not surprising that they are intrinsically disordered proteins (IDPs; Tompa, 2002; Uversky, 2002). IDPs are characterized as proteins that do not maintain stable 2D or 3D structures and instead are better described as having an extended coil structure. In the case of dehydrins, circular dichroism and NMR studies have shown that when the protein is alone in solution, it does not have an overall 3D shape, instead consisting mostly of coil structure, with a weak propensity for helical character in the K-segments (Findlater and Graether, 2009; Ágoston et al., 2011; Hughes and Graether, 2011).

## Localization of Dehydrins to Membranes

Localization of proteins within the cell is often one of the first steps in determining the biological role of proteins. Many studies have shown that dehydrins localize to cellular membranes, suggesting that they could protect them. One of the earliest studies followed dehydrin localization in *Zea mays* using immunogold-labeled antibodies that had been raised against the K-segment (Egerton-Warburton et al., 1997). The gold particles were found dispersed throughout the cytosol, nucleus, but were localized in an uneven manner near membranes. This unevenness was not observed near the endoplasmic reticulum, indicating that they may be preferentially binding some membranes but not others (Egerton-Warburton et al., 1997). Another study on the wheat dehydrin WCOR410 found that it localizes near the plasma membrane in cold-acclimated plants (Danyluk et al., 1998). Under closer inspection, they found that immunogold particles were highly concentrated around the fibrillar network between cell walls, despite not having a known signal peptide for extracellular transport (Danyluk et al., 1998). Taken together, these two studies reveal the potential importance dehydrins in protecting membranes.

More recent studies using subcellular fractionation also provide insight into dehydrins function. The *Solanum sogarandinum* dehydrin DHN24 was also found to be membrane-localized in various tissue types (Szabala et al., 2014). At a subcellular level, immunogold-labeled DHN24 localized to the outer mitochondrial membrane of cold-acclimated phloem cells. The dehydrin was not detected in the mitochondrial component after performing subcellular fractionation, which indicates that DHN24 may bind the outer mitochondrial membrane from the cytosolic side but is not transported into the organelle (Szabala et al., 2014). Cellular fractionation experiments on celery petioles found the calcium-binding, dehydrin-like protein VCaB45 associated with vacuole membranes (Heyen et al., 2002). They treated vacuole membranes with Triton X-100 detergent to show that the protein was bound to the luminal side of the membrane, requiring some sort of import mechanism (Heyen et al., 2002).

Studies on dehydrin localization in transgenic plants enable the researcher to have a greater level of control over experimental conditions and dehydrin type. It has been found that cold acclimation played a role in dehydrin localization to the cell membranes (Puhakainen et al., 2004). Two-thirds of the immunogold-labeled LTI29 were found in the cytosol before acclimation. After cold acclimation, 2/3 of the gold labeling was found on cell membranes and only 1/3 throughout the cytosol, strongly implicating dehydrins in cryoprotection of the plasma membrane (Puhakainen et al., 2004). Another study identified potential protein–protein interactions at the membrane of the pepper dehydrin CaDHN3 (Meng et al., 2021). CaDHN3-GFP fusion protein was localized to the nucleus and cell membrane of *N. benthamiana* leaves. Yeast two hybrid experiments showed that CaDHN3 was found to interact with CaHIRD11, another dehydrin, at the plasma membrane (Meng et al., 2021). A recent bimolecular fluorescent complementation assay demonstrated an association between a dehydrin and aquaporin, a key membrane protein for controlling intracellular water content (Hernández-Sánchez et al., 2019). This opens the possibility of multiple potential dehydrin-dehydrin and dehydrin-membrane protein interactions and may have a role in the mechanism behind membrane stress protection (Hernández-Sánchez et al., 2019; Meng et al., 2021).

## Physiological Protection by Dehydrins

One major complication of freezing stress in plants is the buildup of reactive oxygen species causing lipid peroxidation, which decreases membrane fluidity (Barclay and McKersie, 1994). Two effective methods to gauge membrane damage are measuring malondialdehyde (MDA) levels (MDA is the final product of lipid peroxidation), and measuring electrolyte leakage (EL), since the plasma membrane loses structure under stress, allowing ions to cross the membrane in higher quantities. Conifers represent an interesting model for studying physiological protection from dehydrins because of their natural ability to tolerate extreme cold. It has been observed that the relative electrolyte leakage in cold-acclimated boreal conifers was so low that cells only sustained reversible damage (Strimbeck et al., 2008). To expand upon these results, another study measured dehydrin expression throughout the year in Siberian spruce; Western blot analysis revealed peak dehydrin expression during the winter months when cold protection is needed the most (Kjellsen et al., 2013). The band intensity of dehydrins coincided with T_m_ values, the midpoint monthly temperature; when T_m_ decreased, band intensity increased. In other words, dehydrin expression was highest when the temperature was lowest (Kjellsen et al., 2013). One study measured MDA levels in *Eriobotrya japonica* subjected to freezing stress in the presence of a range of different dehydrins (Xu et al., 2014). Following the stress treatment, MDA levels were 56% higher in freeze intolerant plants than freeze tolerant plants, which is correlated with lower levels of dehydrins in the susceptible cultivar (Xu et al., 2014). This study showed that increased dehydrin expression is implicated in freeze tolerance and decreased levels of oxidative stress.

Dehydrin overproduction in transgenic crops is not just an excellent way to corroborate results seen in native, cold-tolerant plants but is also a promising avenue to extend the growing season and protect plants from extreme weather events. Transgenic strawberry lines overexpressing the wheat dehydrin gene *Wcor410a* were used to test the cryoprotective properties of dehydrins (Houde et al., 2004). The cold-acclimated, transgenic strawberry plants showed 20% EL compared to 60% in the acclimated wild type, and also demonstrated better tolerance to sub-zero temperatures. Consistent with earlier studies performed on transgenic plants, tobacco overexpressing the citrus dehydrin CuCOR19 had decreased levels of EL, indicating less membrane damage than the wild type (Hara et al., 2003). Interestingly, the transgenic tobacco also exhibited faster growth at milder temperatures than the plants not expressing CuCOR19, suggesting that the protective role of dehydrins may not only occur at freezing temperatures. This idea is supported by a study on cultivated tomato, which is known to have few genes related to environmental stress response. *Solanum habrochaites* is a variety of wild tomato that exhibits increased tolerance to the cold (Liu et al., 2015). In the study, they created a transgenic tomato plant by expressing the *S. habrochaites* dehydrin, ShDhn, in the cultivated tomato. Compared to the control wild type, the transgenic lines retained more turgidity after being subjected to a 4°C treatment for 3 days. Following the cold stress, EL of the wild type increased 3.3-fold, while the transgenic lines increased only 2.6-fold. After a 10-day period of drought stress, the transgenic plants retained stem turgidity, while the wild type lost the ability to stand upright (Liu et al., 2015).

## Structural Changes in Dehydrin Upon Membrane Binding

IDPs are not necessarily fully disordered all of the time, many gain structure in the presence of a ligand. This is also the case for dehydrins, and some of the earliest biophysical evidence for a role for dehydrins in protecting membranes came from structural experiments. CD experiments showed that the 35 kDa cowpea dehydrin gained α-helical structure in the presence of SDS micelles (Ismail et al., 1999). These micelles are often used as membrane mimetics because they are easier to use with biophysical techniques while still being a good model system for biological lipids in membranes (Tulumello and Deber, 2009). Subsequent studies have shown that the K-segment is involved in SDS binding and that the gain in α-helicity occurs in these regions (Koag et al., 2009; Atkinson et al., 2016). The deletion of one or two of the K-segments of *Zea mays* DHN1 resulted in less gain in α-helicity and that the deletion of all three K-segments resulted in no structural change (Koag et al., 2009). A later study used NMR to probe the structure of the K_2_ dehydrin in the presence of SDS micelles, obtaining structural information on a per residue basis (Atkinson et al., 2016). This study showed that the K_2_ dehydrin, a protein found in *Vitis riparia* that is a splice variant of the longer YSK_2_ dehydrin, also gained α-helical structure when bound to SDS. Dynamics data from the K_2_ study showed that the bound K-segments were less disordered, while the ϕ-segments (i.e., the poorly conserved region of the dehydrins that connects the conserved segments) retained their flexibility. This supports the idea that the ϕ-segments are highly flexible, even within the context of a disordered protein, and allow for the conserved segments to optimally orient themselves to interact with the ligand(s; Hughes and Graether, 2011).

A more detailed structural analysis (Clarke et al., 2015) using the second structure propensity program δ2Δ (Camilloni et al., 2012) and NMR dynamics experiments showed that the middle of the K-segment was helical nearly 80–90% of the time, while the 3–4 residues flanking these were helical ~20–60% of the time. Extensive modeling of the K_2_-SDS micelle interaction suggested that the Lys residues flank the hydrophobic and negatively charged sides of the K-segment helices, while the few hydrophobic residues of the K-segment are buried near the acyl chains of the membrane (Clarke et al., 2015). A similar result was shown from NMR data using *Arabidopsis thaliana* Lti30 K-segments while bound to small unilamellar vesicle (SUVs, also referred to as liposomes; Eriksson et al., 2016). The center of the peptides was highly helical, though the flanking residues were in an extended, β-strand conformation. The author modeled the interaction between the protein and membrane, and also suggested that the polar and negatively charged face of the K-segment helix are oriented away from the membrane surface (Eriksson et al., 2016).

Other studies have also used SUVs to see whether dehydrins are able to interact with membranes that more closely resemble a biological one. Most studies found that dehydrin largely bound to anionic lipids in SUVs and that an increase in α-helicity was also observed, though at a lower level compared to the proteins in the presence of micelles (Soulages et al., 2003; Koag et al., 2009; Clarke et al., 2015). The requirement for a negative charge on the lipid headgroups likely reflects binding by the Lys residues in the K-segments (Clarke et al., 2015; Atkinson et al., 2016). The ionization state of the di-His sequences that flank some K-segments are also important for binding (Eriksson et al., 2011). Using the Lti30 dehydrin, they showed that the deprotonation of the His residues prevented binding of the protein to SUVs. These His residues are not part of the K-segment helix but may be positioning themselves so that they interact with the amines on the head groups (Eriksson et al., 2011).

The need for anionic headgroups appears to depend on the dehydrin and the lipids being used in the study. In several of the studies reported above, it was shown that SUVs consisting only of phosphatidylcholine (PC) did not bind dehydrins (Soulages et al., 2003; Koag et al., 2009; Clarke et al., 2015). PC is zwitterionic, but the presence of the negative phosphate in the headgroup did not appear to be sufficient to allow for binding as measured by CD experiments or SUV pulldown assays. However, other dehydrin studies appear to contradict either the change in structure or the need for acidic lipids. Experiments using ERD10 and ERD14, dehydrins found in *Arabidopsis thaliana*, did not show a change in the CD spectra in the presence of acidic SUVs (i.e., there was no evidence of a gain in α-helicity), yet mini-gel filtration assays suggest that the proteins did interact (Kovacs et al., 2008). Likewise, using surface plasmon resonance, an interaction was also detected between Lti30 and PC SUVs (Eriksson et al., 2011). Whether these results come from a specific feature of these dehydrins, or whether detection depends on the exact experimental conditions and measuring equipment used, will require further studies.

## Mechanistic Evidence for Membrane Protection

**Figure 1:**
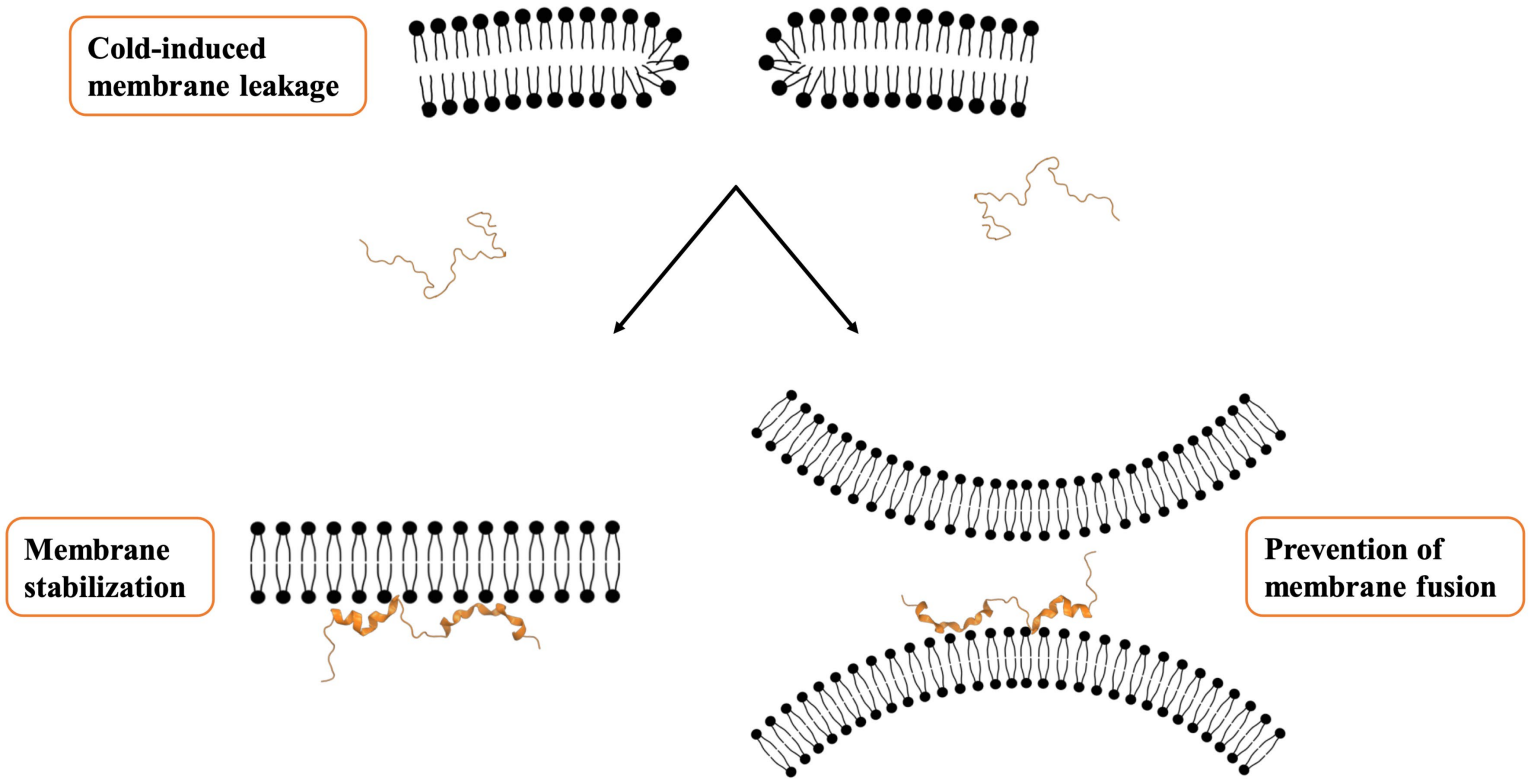
Proposed mechanism for membrane protection. The binding of dehydrins to a biological membrane appears to have two protective effects. One is to maintain membrane fluidity at low temperatures, while the second is to prevent membranes from fusing, either by maintaining the fluidity or by helping to keep them spaced apart.

In addition to monitoring structural changes in dehydrins, biophysical experiments also allow one to explore the mechanisms by which dehydrins exert their protective role on membranes (Figure 1). Studies have brought protective mechanisms to light, with two effects being reported; one is the decrease in the membrane transition temperature (T_m_; Clarke et al., 2015), and the other a lowering of the relative humidity (R_h_) at which the transition to hexagonal (II) phase occurs, where the membrane forms an inverted micelle structure (Andersson et al., 2020). Both share the common effect of maintaining membrane fluidity and keep it functional despite changes in temperature or water content. In the study with the *Vitis riparia* K_2_, it was also found that the protein was able to prevent the fusion of liposomes after a freeze/thaw stress was applied (Clarke et al., 2015). Specifically, there was a correlation between the reduction in the average size of the fused SUVs (as assessed by dynamic light scattering) and the concentration of K_2_. The use of PEG3350 (a polymer with a similar hydrodynamic radius to K_2_) did not prevent fusion, while the addition of K-segment peptides alone (i.e., a dehydrin without its ϕ-segments) did prevent it. These two control experiments suggest that the dehydrins prevent fusion not by steric hinderance but by altering the fluidity of the membrane through a direct interaction. A recent study showed that K-segments alone were also able to prevent aggregation of liposomes and that removing any of the charged or hydrophobic residues in the sequence drastically inhibited this protective function (Kimura et al., 2022). While membrane fusion was not directly examined, a different dehydrin (Lti30) using another assay showed that these proteins were able to cluster SUVs. Their interpretation of the role of the dehydrin is to maintain a consistent separation of membranes, where one K-segment in Lti30 would bind one membrane, while another K-segment could bind another membrane (Eriksson et al., 2016). As water content might vary, for example, during cold stress, these dehydrins would provide the structure needed to keep membrane integrity and prevent them from rupturing. In this model, the ϕ-segments could be extended so as to facilitate the “cross-linking” of two different SUVs.

## Conclusion

Dehydrin-membrane binding plays a fundamental role in plant cryoprotection, as membranes are one of the primary sites of cold-induced damage. One issues that need resolving is the multiple protective functions of dehydrins in addition to membrane protection (e.g., enzyme protection and protecting DNA from reactive oxygen species). While moonlighting has shown that IDPs can have multiple functions, why a dehydrin can switch between membrane protection at the membrane, enzyme protection in the cytosol, and DNA protection in the nucleus is not yet clear. In addition, the exact mechanism by which cryoprotection occurs will need further study, because different effects have been described by various studies. Dehydrins can reduce the amount of membrane fusion, while they can also promote membrane clustering. Two different protective mechanisms have also been proposed as: keeping membranes fluid versus keeping them consistently separated. To answer, all of these questions may require studies with *in vivo* systems, where there will be direct evidence of dehydrin cryoprotection and membrane binding in the cell.

## Author Contributions

MM and SG wrote and revised the manuscript. All authors contributed to the article and approved the submitted version.

## Funding

This study was supported by NSERC Discovery Grant to SG (2016–04253).

## Conflict of Interest

The authors declare that the research was conducted in the absence of any commercial or financial relationships that could be construed as a potential conflict of interest.

## Publisher’s Note

All claims expressed in this article are solely those of the authors and do not necessarily represent those of their affiliated organizations, or those of the publisher, the editors and the reviewers. Any product that may be evaluated in this article, or claim that may be made by its manufacturer, is not guaranteed or endorsed by the publisher.
